# Quantified treatment effect at the individual level is more indicative for personalized radical prostatectomy recommendation: implications for prostate cancer treatment using deep learning

**DOI:** 10.1007/s00432-023-05602-4

**Published:** 2024-02-01

**Authors:** Huiqing Pan, Jiayi Wang, Weizhong Shi, Ziqin Xu, Enzhao Zhu

**Affiliations:** 1 School of Medicine, Tongji University, Shanghai, China; 2 Shanghai Hospital Development Center, Shanghai, China; 3 Columbia University, New York, USA

**Keywords:** Prostate cancer, Prostatectomy, Deep learning, Causal inference

## Abstract

**Background:**

There are potential uncertainties and overtreatment existing in radical prostatectomy (RP) for prostate cancer (PCa) patients, thus identifying optimal candidates is quite important.

**Purpose:**

This study aims to establish a novel causal inference deep learning (DL) model to discern whether a patient can benefit more from RP and to identify heterogeneity in treatment responses among PCa patients.

**Methods:**

We introduce the Self-Normalizing Balanced individual treatment effect for survival data (SNB). Six models were trained to make individualized treatment recommendations for PCa patients. Inverse probability treatment weighting (IPTW) was used to avoid treatment selection bias.

**Results:**

35,236 patients were included. Patients whose actual treatment was consistent with SNB recommendations had better survival outcomes than those who were inconsistent (multivariate hazard ratio (HR): 0.76, 95% confidence interval (CI), 0.64–0.92; IPTW-adjusted HR: 0.77, 95% CI, 0.61–0.95; risk difference (RD): 3.80, 95% CI, 2.48–5.11; IPTW-adjusted RD: 2.17, 95% CI, 0.92–3.35; the difference in restricted mean survival time (dRMST): 3.81, 95% CI, 2.66–4.85; IPTW-adjusted dRMST: 3.23, 95% CI, 2.06–4.45). Keeping other covariates unchanged, patients with 1 ng/mL increase in PSA levels received RP caused 1.77 months increase in the time to 90% mortality, and the similar results could be found in age, Gleason score, tumor size, TNM stages, and metastasis status.

**Conclusions:**

Our highly interpretable and reliable DL model (SNB) may identify patients with PCa who could benefit from RP, outperforming other models and clinical guidelines. Additionally, the DL-based treatment guidelines obtained can provide priori evidence for subsequent studies.

**Supplementary Information:**

The online version contains supplementary material available at 10.1007/s00432-023-05602-4.

## Introduction

Prostate cancer (PCa) is a common cancer in men aged 65 or above, causing substantial mortality and morbidity worldwide. It is estimated that nearly 1.3 million people are newly diagnosed worldwide every year, and approximately 400,000 suffer from treatment-related morbidity (Global regional and national incidence prevalence 2017; Foreman et al. 2018). Although various therapies and management of both primary and metastatic PCa have advanced rapidly (Sandhu et al. 2021), it is still difficult to balance treatments and risks of progression with therapy-related health problems (Donovan et al. 2016). This may imply that we should focus on recognizing those who can benefit from specific therapies.

Radical prostatectomy (RP) has been considered a standard treatment for TNM stage I–III PCa patients (Sekhoacha et al. 2022). It could prevent further metastatic seeding and late complications of aggressive PCa (Costello 2020). RP was widely applied to patients with low-risk PCa rather than those with high-risk PCa in the 1980s and 1990s (Costello 2020). However, over the past 40 years, the role of RP in treating prostate cancer has changed considerably because of RP’s significant risk of overtreatment and accompanying adverse effects (Hamdy et al. 2023). For example, a shift in the application of RP toward PCa patients with high risks occurred, and the survival time was similar in low-risk PCa patients who received RP or other therapies (Wilt et al. 2017, 2020). A review also found that RP was related to decreased cancer-specific quality of life (Lardas et al. 2017) partly due to RP’s effect on biological functions (Litwin and Tan 2017), indicating that it might not always be appropriate to use RP. As such, identification of optimal candidates for RP is quite important to make PCa patients benefit more from therapies and avoid overtreatment.

Therefore, this study aims to establish a model to discern whether an individual patient can benefit more from RP and to identify heterogeneity in treatment responses among PCa patients.

## Materials and methods

### Study design

All patients were included from the Surveillance, Epidemiology, and End Results (SEER) database, which comprises data from 18 regions across the United States, accounting for approximately 30% of the national population (Islami et al. 2021). This study adhered to the Strengthening the Reporting of Observational Studies in Epidemiology reporting guidelines for observational research (Elm et al. 2007).

Men aged 18 or above who were diagnosed with PCa as a primary cancer and who received RP or did not undergo surgery between 2010 and 2017 were included. Anatomic site codes (C61.9) and histology subtypes (8140) were classified according to the International Classification of Disease for Oncology, 3rd edition. We excluded those falling under any of the following:

1. Age below 18;
2. Lack of clear data on Gleason scores, TNM stage, clinical prostate-specific antigen (PSA) level, or tumor size;
3. Unknown demographic information;
4. Unknown survival months;
5. Unknown metastasis status.

**Fig. 1:**
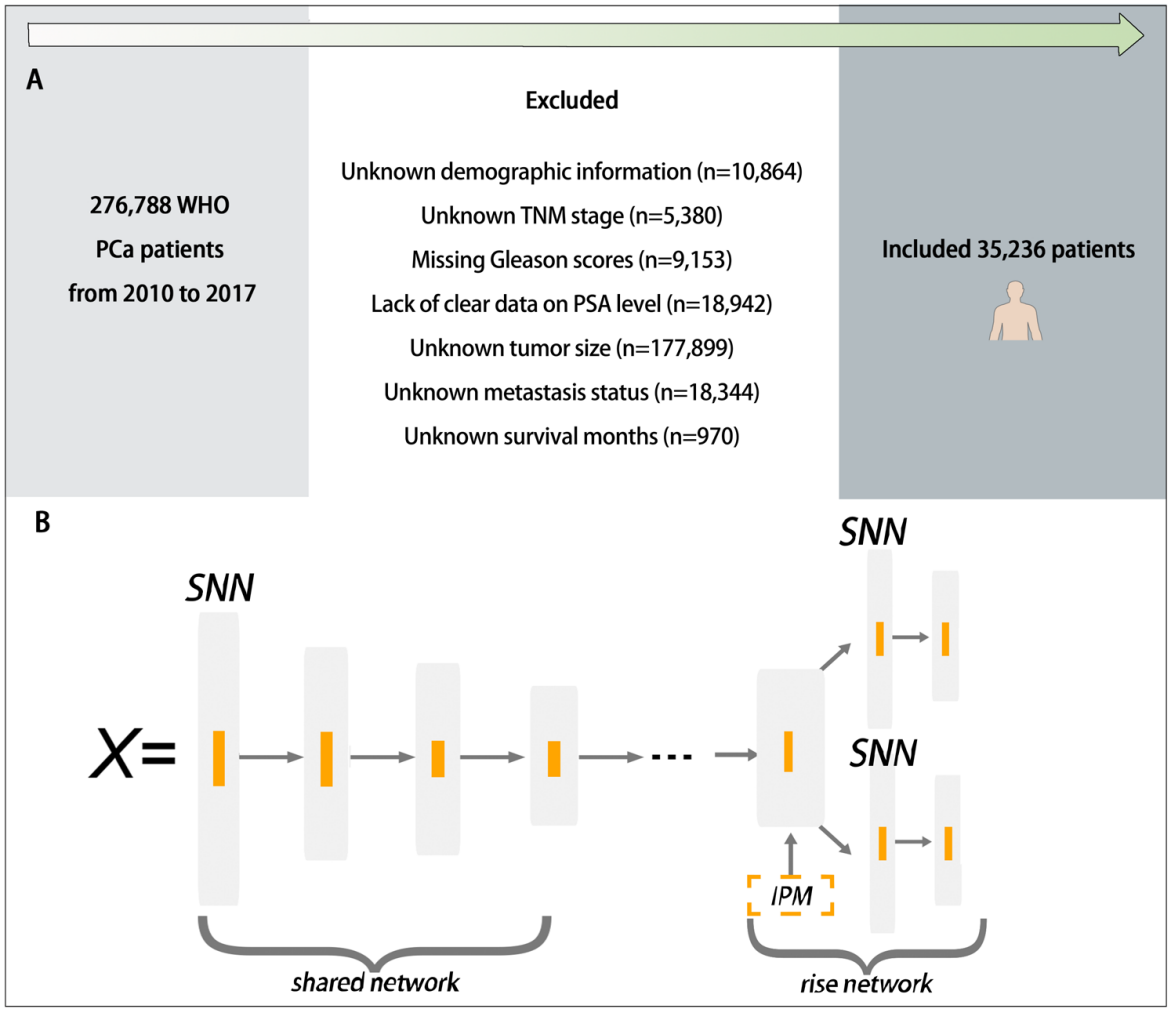
Flowchart of patient inclusion and schematic of the SNB architecture. **A** Flowchart of patient inclusion. **B** Schematic of the SNB architecture. *PCa* prostate cancer; *PSA* prostate-specific antigen; *SNN* Self-normalizing neural network; *IPM* integral probability metrics

Figure 1A provides a comprehensive illustration of the participant inclusion process. The focal outcome under examination was overall survival (OS), a metric provided by SEER, denoting the time period between all-cause death and the initial PCa diagnosis. Patients who were still alive in December 2020 were considered censored data, so the minimum follow-up time was 3 years.

### Deep learning algorithms

T-learner is a commonly used model for inferring the individual treatment effect (ITE)(Künzel et al. 2019), which trains each base model on different intervention groups separately and obtains the conditional average treatment effect (CATE). However, T-learner has some drawbacks: (1) it relies heavily on the performance of the trained base models, whose performance can be affected by extremely imbalanced training numbers in two groups (Yao et al. 2020); (2) the ignorability of T-learner only eliminates confounding artifacts, while imbalances in generating distributions due to biased treatment allocation could still be present.

Benefitting from the development of deep learning (DL) and representation learning, balancing the generating distributions of the different treatment groups has been proven to be effective for both covariate space (Li et al. 2014) and latent representations (Johansson et al. 2020). Balanced Individual Treatment Effect for Survival data (BITES) (Schrod et al. 2022), a semiparametric survival regression DL model, contains a shared network and two risk networks. In a shared network, balanced generating distributions are obtained by using integral probability metrics (IPM) to maximize the p-Wasserstein distance of the latent representations of different treatment arms, and the smoothed optimal transport loss is calculated, which is called representation-based causal inference.

Self-normalizing neural networks (SNNs) enhanced feed-forward neural networks (FNNs) and significantly outperformed all competing FNN methods (Klambauer et al. 2017). The neuron activations of SNNs automatically converge toward zero mean and unit variance, which in turn avoids exploding and vanishing gradients. Thus, in this study, we introduce the Self-Normalizing Balanced individual treatment effect for Survival data (SNB). SNB inherits the architecture of BITES, while scaled exponential linear units (SELUs) are added in both shared networks and risk networks. Shared network contains a five-layered SNN with a dropout rate of 10%. The shared network receives input features and uses IPM to balance the latent representations between each treatment group. Risk networks are two identical four-layered SNNs, which, respectively, represent the regularized representations of Non-Surgery group and RP group. At time of inference, the SNB calculates the corresponding treatment-specific baseline hazards in each of the two risk networks. By varying the risk networks which an individual's features are entered and its baseline hazards, SNB can predict survival outcomes under the hypothesis of different treatments, thereby visualizing the advantages and disadvantages of different treatments. The architecture of SNB is presented in Fig. 1B.

### Model development and treatment recommendation

Temporal validation(Cooray et al. 2023) was utilized to validate models. We allocated patients diagnosed from 2010 to 2015 (24,464 (69.4%) patients) to a training set that was used for building the models and a testing set which consists of patients diagnosed from 2016 to 2017 (10,772 (30.6%) patients) to evaluate the models’ performance and the effect of the models’ recommendation. During the training period, we used fivefold cross-validation to tune the model hyperparameters. The training process was terminated automatically if the validation loss did not decrease in 1,000 iterations. We trained SNB, BITES, Cox Mixtures with Heterogeneous Effects (CMHE) (Nagpal et al. 2022), DeepSurv (Katzman et al. 2016), Cox proportional hazards model (CPH), and random survival forest (RSF). CMHE, DeepSurv, CPH, and RSF were trained and used in the form of T-learner.

In estimating the individual treatment effect (ITE), only one fact can be observed per patient, and the outcome of the alternative scenario is unobservable. Thus, these outcomes need to be predicted by models. The individual survival distribution is obtained with the predicted log hazard ratios and treatment-specific baseline hazards, which describes the change in survival probability over time. We defined the outcome as the time it took for an individual patient to reach 90% mortality under the predicted individual survival distribution, called the time at risk (TaR). The TaR represents the time interval between PCa diagnosis and the time when his mortality rate reaches 90%. The ITE is therefore calculated as $ITE={TaR}^{T=1}-{TaR}^{T=0}$, where $T=1$ represents the situation in which the patient receives RP and $T=0$ represents the situation in which he does not receive the procedure. In such cases, an individual patient was recommended for RP or non-surgery based on whether the ITE was greater than zero.

The ITE calculation methods of all models were identical. To explore the recommendation effect of the models, we divided the patients into the recommended (Consis.) and anti-recommended (Inconsis.) groups, based on whether the actual treatment they received was consistent with the model recommendations.

### Statistical analyses

Statistical analyses were performed using R 4.1.3 and Python 3.8. Continuous variables are reported as medians and interquartile ranges (IQRs), and categorical variables are presented as numbers and percentages (%). Inverse probability treatment weighting (IPTW) was used to avoid treatment selection bias. The log-rank test was used to compare Kaplan–Meier (KM) curves.

## Results

### Study population

A total of 35,236 PCa patients with complete follow-up records who met the inclusion criteria were included in this study. The overall mortality rate was 6.3% (95% CI: 6.1%–6.6%) over a median (IQR) follow-up time of 76 (52–106) months. The median (IQR) age was 63 (57–68) years, and the median (IQR) tumor size was 17 (11–24) mm. A total of 6,265 (17.8%) patients were in the Non-Surgery group, while 28,971 (82.2%) underwent radical RP. The baseline clinical characteristics of all patients are presented in Table 1.

**Table 1:** Baseline demographic and pathological features

	Surgery (*n* = 28,971)	Non-Surgery (*n* = 6265)
Age, median (IQR), y	62 (57–67)	68 (62–73)
Tumor size, median (IQR), mm	17 (12–24)	14 (8–25)
Married	22,180 (76.6)	4165 (66.5)
Race––White	23,173 (80.0)	4885 (78.0)
Income––Higher than 70,000$	14,082 (48.6)	3,645 (58.2)
Region––Urban	26,100 (90.1)	5714 (91.2)
Grade		
I	1509 (5.2)	1283 (20.5)
II	13,252 (45.7)	2,540 (40.5)
III	14,191 (49.0)	2,4331 (38.8)
IV	19 (0.1)	11 (0.2)
T stage		
T1	94 (0.3)	3210 (51.2)
T2	19,851 (68.5)	2226 (35.5)
T3	8934 (30.8)	653 (10.4)
T4	86 (0.3)	175 (2.8)
N stage		
N0	27,708 (95.6)	5896 (94.1)
N1	1257 (4.3)	368 (5.9)
M stage		
M0	28,893 (99.7)	5860 (93.5)
M1	72 (0.2)	404 (6.4)
TNM stage		
I	1434 (4.9)	1684 (26.9)
IIA	2666 (9.2)	2092 (33.4)
IIB	15,670 (54.1)	1389 (22.2)
III	7,850 (27.1)	474 (7.6)
IV	1351 (4.7)	626 (10.0)
Distant metastasis		
Bone	53 (0.2)	353 (5.6)
Brain	0 (0.0)	4 (0.1)
Liver	0 (0.0)	15 (0.2)
Lung	1 (< 0.1)	27 (0.4)
Lymph node-removed		
None	9982 (34.5)	6243 (99.6)
1 ≤ Num ≤ 3	5000 (17.3)	5 (0.1)
≥ 4	13,789 (47.6)	17 (0.3)
Prostate-specific antigen, median (IQR), ng/mL	6.2 (4.8–9.2)	7.6 (5.2–13.1)
Primary Gleason Score		
1	1 (< 0.1)	0 (0.0)
2	7 (< 0.1)	8 (0.1)
3	19,453 (67.1)	3725 (59.5)
4	9064 (31.1)	2195 (35.0)
5	446 (1.5)	337 (5.4)
Secondary Gleason Score		
1	1 (< 0.1)	0 (0.0)
2	15 (0.1)	9 (0.1)
3	14,145 (48.8)	3067 (49.0)
4	13,137 (45.3)	2490 (39.7)
5	1673 (5.8)	699 (11.2)
Survival time, median (IQR), month	80 (55–108)	58 (43–90)
Prostate cancer-specific mortality	338 (1.2)	350 (5.6)
Overall mortality	1284 (4.4)	948 (15.1)

### Model performance

CPH achieved the best discrimination (integrated Brier score in Non-Surgery group (IBS^a^): 0.09, 95% CI, 0.08–0.10; integrated Brier score in RP group (IBS^b^): 0.04, 95% CI, 0.03–0.04), followed by SNB (IBS^a^: 0.10, 95% CI, 0.09–0.12; IBS^b^: 0.04, 95% CI, 0.03–0.04).

We calculated the multivariate hazard ratio (HR), the difference in restricted mean survival time (dRMST) (month), and risk difference (RD) (%) to evaluate the protective effect of each model. HR describes the multiplicity of changes in mortality resulting from following model recommendations. dRMST describes the average additional survival time of patients in the Consis. compared to the Inconsis. group. RD describes absolute mortality reductions resulting from following model recommendations. To avoid imbalances of prognostic factors between the Consis. and Inconsis. groups, we used IPTW to correct for the above metrics, in which covariates, including age, tumor size, histological grades, TNM stages, metastatic sites, lymph node involvements, PSA level, and Gleason scores, were corrected. In such cases, these metrics were expected to reflect the debiased treatment recommendation performance. All metrics were calculated based on overall survival (OS) with a time horizon of 10 years. The detailed model performance is presented in Table 2.

**Table 2:** Detailed model performance and treatment recommendation effect

Model	IBS^a^	IBS^b^	HR	HR^c^	RD (%)	RD^c^ (%)	dRMST (month)	dRMST^c^ (month)
SNB	0.10 (0.09–0.11)	0.04 (0.03–0.05)	0.76 (0.64–0.92)	**0.77 (0.61–0.95)**	3.80 (2.48–5.11)	2.17 (0.92–3.35)	3.81 (2.66–4.85)	**3.23 (2.06–4.45)**
BITES	0.12 (0.11–0.12)	0.05 (0.05–0.06)	0.71 (0.58–0.84)	0.56 (0.23–1.34)	8.15 (6.26–10.05)	6.33 (4.16–8.52)	8.49 (6.74–10.24)	2.98 (1.60–4.28)
CMHE	0.14 (0.13–0.15)	0.07 (0.07–0.07)	0.74 (0.60–0.88)	1.67 (0.91–3.10)	**8.62 (6.62–10.62)**	6.48 (4.25–8.71)	**9.08 (7.22–10.94)**	-12.38 (-14.48–-10.68)
DeepSurv	0.22 (0.19–0.23)	0.29 (0.28–0.28)	0.96 (0.78–1.19)	0.92 (0.41–2.10)	-4.93 (-6.72–-3.15)	-4.45 (-6.52–-2.39)	-5.27 (-6.94–-3.61)	-10.15 (-12.39–-8.31)
CPH	**0.09 (0.09–0.10)**	**0.04 (0.03–0.05)**	0.83 (0.69–0.97)	0.85 (0.67–1.06)	3.39 (2.16–4.51)	3.52 (2.49–4.76)	3.22 (2.15–4.26)	-8.56 (-9.77–-7.34)
RSF	0.10 (0.10–0.11)	0.05 (0.04–0.06)	**0.70 (0.58–0.84)**	0.57 (0.24–1.33)	8.17 (6.27–10.05)	**6.54 (4.43–8.65)**	8.47 (6.73–10.24)	2.96 (1.34–4.27)
NCCN	.	.	0.87 (0.72–1.07)	1.06 (0.79–1.40)	5.19 (3.49–4.83)	5.38 (2.98–7.78)	6.88 (5.17–8.58)	2.02 (0.67–3.64)

All metrics are calculated based on a 10-year time horizon. Bolded font indicates that the model performs best in this metric. NCCN, National Comprehensive Cancer Network. According to 2023 NCCN Guideline, risk groups are classified into 6 categories: Very low, Low, Favorable intermediate, Unfavorable intermediate, High, and Very high, among which intermediate, High risk, and M0 stage plus N1 stage are recommended for radical prostatectomy. Patients whose actual treatment was consistent with the NCCN recommendation were compared with those who were inconsistent

*SNB* Self-Normalizing Balanced individual treatment effect for survival data; *BITES* Balanced Individual Treatment Effect for Survival data; *CMHE* Cox Mixtures with Heterogeneous Effects; *CPH* Cox proportional hazards model; *RSF* random survival forest

IBS^a^, integrated Brier score in the Non-Surgery group; IBS^b^, integrated Brier score in the prostatectomy group; HR, multivariate hazards ratio; RD, risk difference; dRMST, the difference in restricted survival time; ^c^, adjusted for all covariates using inverse probability treatment weighting

Among all models, only SNB achieved the best IPTW-adjusted HR (HR^c^) and IPTW-adjusted dRMST (dRMST^c^) (HR: 0.76, 95% CI, 0.64–0.92; HR^c^: 0.77, 95% CI, 0.61–0.95; RD: 3.80, 95% CI, 2.48–5.11; IPTW-adjusted RD (RD^c^): 2.17, 95% CI, 0.92–3.35; dRMST: 3.81, 95% CI, 2.66–4.85; dRMST^c^: 3.23, 95% CI, 2.06–4.45). CMHE had the best RD and dRMST (HR: 0.74, 95% CI, 0.60–0.88; HR^c^: 1.67, 95% CI, 0.91–3.10; RD: 8.62, 95% CI, 6.62–10.62 RD^c^: 6.48, 95% CI, 4.25–8.71; dRMST: 9.08, 95% CI, 7.22–10.94; dRMST^c^: -12.38, 95% CI, -14.48–-10.68); BITES had the best HR (HR: 0.71, 95% CI, 0.58–0.84; HR^c^: 0.56, 95% CI, 0.23–1.34; RD: 8.15, 95% CI, 6.26–10.05; RD^c^: 6.33, 95% CI, 4.16–8.52; dRMST: 8.49, 95% CI, 6.74–10.24; dRMST^c^: 2.98, 95% CI, 1.60–4.28); and RSF had the best RD^c^ (HR: 0.70, 95% CI, 0.58–0.84; HR^c^: 0.57, 95% CI, 0.24–1.33; RD: 8.17, 95% CI, 6.27–10.05; RD^c^: 6.54, 95% CI, 4.43–8.65; dRMST: 8.47, 95% CI, 6.73–10.24; dRMST^c^: 2.96, 95% CI, 1.34–4.27). However, no model, except for SNB, can achieve a statistically significant HR^c^.

We compared the 2023 National Comprehensive Cancer Network (NCCN) guideline(Schaeffer et al. 2022). Patients whose actual treatment was consistent with the NCCN recommendation were compared with those who were inconsistent. However, the protective effect of NCCN recommendation (HR: 0.87, 95% CI, 0.72–1.07; HR^c^: 1.06, 95% CI, 0.79–1.40; RD: 5.19, 95% CI, 3.49–4.83; RD^c^: 5.38, 95% CI, 2.98–7.78; dRMST: 6.88, 95% CI, 5.17–8.58; dRMST^c^: 2.02, 95% CI, 0.67–3.64; *P* of log-rank test < 0.001; *P* of IPTW-adjusted log-rank test = 0.240) was inferior to our best model, SNB, particularly on multivariate and IPTW-adjusted metrics.

**Fig. 2:**
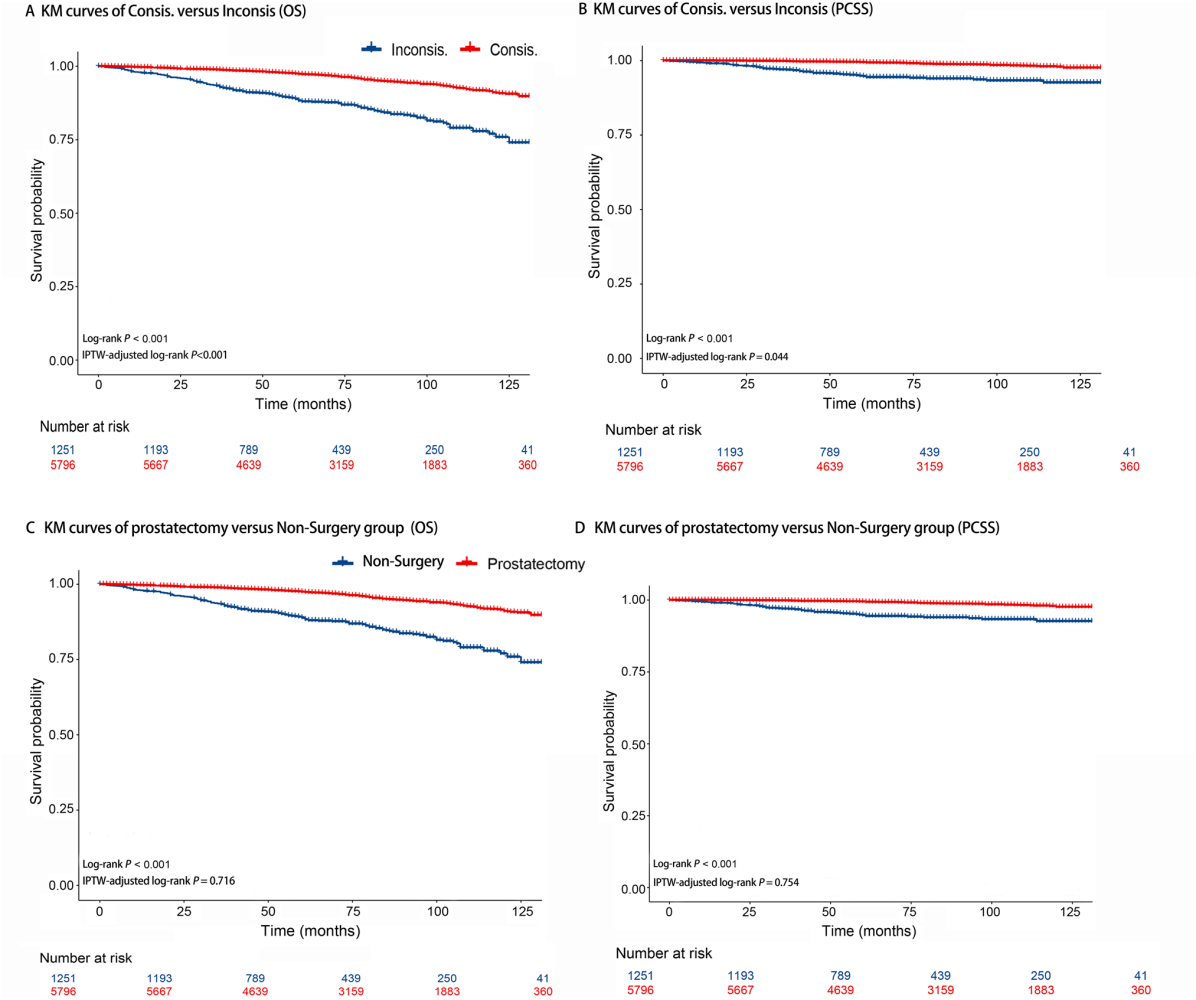
KM curves comparison. **A** KM curves of Consis. versus Inconsis. regarding overall survival. **B** KM curves of Consis. versus Inconsis. regarding prostate cancer-specific survival. **C** KM curves of radical prostatectomy versus Non-Surgery group regarding overall survival. **D** KM curves of radical prostatectomy versus Non-Surgery group regarding prostate cancer-specific survival. *IPTW* inverse probability treatment weighting; *OS* overall survival; *PCSS*, prostate cancer-specific survival

We present the KM curves of Consis. versus Inconsis. regarding OS and prostate cancer-specific survival (PCSS) in Fig. 2A and B, respectively. Better OS outcomes (*P* of log-rank test < 0.001; *P* of IPTW-adjusted log-rank test < 0.001) and PCSS outcomes (*P* of log-rank test < 0.001; *P* of IPTW-adjusted log-rank test = 0.044) were observed. Figure 2C and D shows the KM curves of RP versus Non-Surgery group for OS and PCSS. The OS (*P* of log-rank test < 0.001) and PCSS (*P* of log-rank test < 0.001) advantages of RP were observed; however, this advantage no longer existed after IPTW correction (*P* of IPTW-adjusted log-rank test of OS = 0.716; *P* of IPTW-adjusted log-rank test of PCSS = 0.754).

**Fig. 3:**
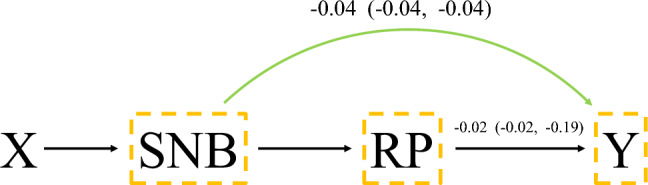
Causal path of SNB recommendations. *SNB* Self-Normalizing Balanced individual treatment effect for survival data; RP, radical prostatectomy; X indicates the covariates of patients. Y indicates patients' mortality

In addition, we presented the causal path of the protective effect of SNB in Fig. 3. RP was treated as a mediator variable, while all covariates were treated as potential confounders. Thus, the natural direct effect (NDE) and natural indirect effect (NIE) of SNB recommendation was calculated. These values were presented as the slope of a linear regression. After excluding the effect of RP, the protective effect of SNB remained statistically significant (NDE: – 0.04, 95% CI, – 0.04–-0.04).

### The deep learning-based treatment guidelines

**Fig. 4:**
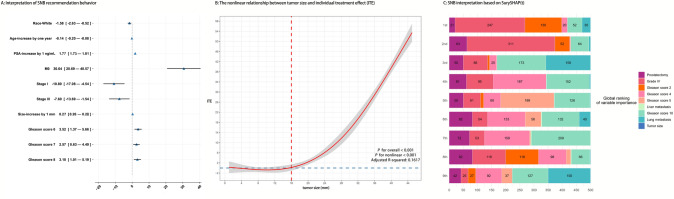
Model interpretation. **A** Interpretation of SNB recommendation behavior. **B** The non-linear relationship between tumor size and individual treatment effect. **C** SNB interpretation based on SurvSHAP(t). The individual treatment effect measures the time it took for a patient to reach 90% mortality receiving radical prostatectomy

To explain the recommendation behavior of SNB, we derived a mixed effect linear regression that predicts ITE from the covariates. Household income and reporting region were set as random effects. Thus, the beta values obtained indicate the presence of this covariate or an increase of one that causes the difference in the time it took for the patient to reach 90% mortality of RP over no surgery to increase beta. This result is presented in Fig. 4A.

RP was more effective in patients with higher PSA levels (1.77, 95% CI, 1.73–1.81), non-metastatic disease (30.64, 95% CI, 20.69–40.57), larger tumor size (0.27, 95% CI, 0.26–0.28), Gleason score 6 (3.52, 95% CI, 1.37–5.66), Gleason score 7 (2.57, 95% CI, 0.63–4.49), and Gleason score 8 (3.10, 95% CI, 1.01–5.19). Older ( – 0.14, 95% CI,  – 0.20–  – 0.08), white-raced ( – 1.58, 95% CI,  – 2.63– – 0.52), TNM stage I (-10.80, 95% CI,  – 17.08  – 4.54), and stage III ( – 7.60, 95% CI, – 13.69– – 1.54) patients were not optimal candidates for RP.

In addition, we used the restricted cubic spline model to assess the non-linear relationship between tumor size and ITE, which is presented in Fig. 4B. The optimal knot was tested between 3 and 5 using R^2^. Patients with tumors larger than 16 mm were found to benefit from RP (ITE > 0).

### Model interpretation based on SurvSHAP(t)

We used SurvSHAP(t) to interpret the functional output of SNB, which is the first method introduced to date that can provide a time-dependent interpretation with a solid theoretical basis (Krzyzi’nski et al. 2022). Figure 4C visualizes the aggregation of the eight most important variables, sorted by aggregated Shapley values, rankings over 500 observations. The horizontal bars represent the number of observations where the importance of the variable is ranked first, second, and so on, indicated by the given color. It should be noted that RP in SNB is treated through different risk networks and using different baseline hazards rather than a routine variable. Histological grade was deemed the most important prognostic factor in 247 samples. In addition, Gleason scores and metastasis sites were both important.

To evaluate the importance of features, Table S1 visualizes the changes of overall IBS, IBS^a^, and IBS^b^ of SNB after excluding the eight most important variables in the testing set, whose conclusions are essentially the same as the findings of SurvSHAP(t).

## Discussion

PCa is the most common non-skin cancer in men and ranks second in cancer-related death in the United States, causing 76,234 deaths in all ages in 2018 (Siegel et al. 2021). Although advances in treatment and earlier stage diagnosis continue to emerge (Luh et al. 2018), these treatments are not yet widely used in clinical practice, which highlights the need of constructing an individualized RP recommendation system to extend life expectancy. Therefore, we introduced and carefully evaluated SNB in this study, which outperformed recently proposed or widely used models, real-world physician choices, and NCCN guideline. After adjusting for confounders, SNB led to 6% reduction in patient mortality. Treatment selection often needs to consider complex feature interactions rather than being based on fixed guidelines, and our study demonstrated that DL models are well suited to accomplish this, as clearly evidenced by the stronger protective effect of SNB than NCCN guideline. We hypothesized that there might be other factors that influence treatment choice, not found by existing studies. DL models can identify this complex, potential interaction, embodying the rationality and reliability of SNB.

We believed that the superiority of SNB is attributed to the better predictive power and stability of SNNs over FNNs and single-layer linear regression. On the drug discovery benchmark, SNNs have outperformed other FNNs with and without normalization techniques, such as batch, layer, and weight normalization, or specialized architectures, such as Highway (Zilly et al. 2016) or Residual networks (Klambauer et al. 2017; Xie et al. 2017). It has been proven that SNNs do not face vanishing and exploding gradient problems (Klambauer et al. 2017), which may explain the better performance of SNB compared to BITES.

The nature of artificial intelligence-guided intervention studies gives us the opportunity to obtain DL-based treatment guidelines by interpreting the treatment recommendation behavior of the model. We considered and excluded the influence of confounders on treatment recommendations by holding other parameters unchanged. Consistent with previous studies, we found that baseline features like age (Mottet et al. 2021) worked together with tumor characteristics including TNM stages (Miao et al. 2023), PSA level (Drobner et al. 2023), and Gleason scores (Lam et al. 2019a) significantly affect RP selection, owing to the fact that they are essential factors in life expectancy (Daskivich 2015). However, our models quantified those elements in detail. We found that patients with 1 ng/mL increase in PSA levels receiving RP caused 1.77 months increase in the time to 90% mortality, and the similar results could be found in age, Gleason Score, tumor size, TNM stages, and metastasis status.

Another crucial finding of our research is that 16 mm is the recommended value of tumor size for RP. Exact tumor size indicator for RP was not unified by available evidence (e.g., some chose 5 mm as the critical value for selection of RP (Zhou et al. 2021), while others recommend 10 mm (Sanguedolce et al. 2018; Lam et al. 2019b)), which was considered as the demonstration of disease stratification and prognosis. Our finding was generally inconsistent with Zhou et al.’s conclusion (Zhou et al. 2021). We hypothesized that this result may attribute to the improvement of modern imaging and treatment efficacy that gave opportunities to early intervene small-size tumors. By applying multiparametric magnetic resonance imaging, Park et al. pointed that tumor size ≥ 15 mm was significantly associated with adverse pathology (Baboudjian et al. 2023), which was similar with us and deserve to be further investigated. Based on this situation, DL might provide a new potential for the suggestion of exact tumor size.

Our model (SNB) may serve as a useful analytical tool for treatment recommendation in patients with PCa, given its evidence of the significant prognostic benefits of following the treatment recommendation, which clearly outweigh those associated with not following the recommendation. It is a surgeon’s duty to introduce clinical information to patients. To facilitate discussion of different potential surgical options, surgeons and patients need an informative tool that focuses on survival benefits. In real cases, the establishment of a treatment recommendation system based on a DL model will be key to effectively conveying results and illustrating complex analyses, including prognostic prediction, treatment recommendation to patients and family members, and improving the surgeons’ understanding of the treatment benefits (Wang et al. 2019; Simon et al. 2019; Zhu et al. 2023).

This study has several inevitable limitations. The SEER database did not include information about comorbidities and details of gene panels, which are important for RP selection. Second, although the OS outcome is critical for therapy decisions, the individual preferences of PCa patients and surgeons may reduce the applicability of the model. Third, since OS was the focal outcome, we did not analyze other outcomes, such as quality of life and progression-free survival. Finally, subsequent studies are encouraged to continue to validate the SNB in real-world cohorts to ensure its reliability in clinical practice. Therefore, it remains more various data to maximize the efficacy of models.

## Conclusion

In conclusion, SNB successfully predicted which patients with PCa would benefit from receiving RP. The DL-based treatment guidelines were generally consistent with clinical knowledge and may provide priori evidence for subsequent studies. Subsequent studies are needed to further analyze more comprehensive clinical data. DL models have the potential to obtain information with complex heterogeneity of real-world practice and to recommend treatment precisely for individual PCa patients.

## Supplementary Information

Below is the link to the electronic supplementary material.

Supplementary file1 (DOCX 16 KB)

## Data Availability

This study analyzed public datasets that can be found here: the Surveillance, Epidemiology, and End Results Program (https://seer.cancer.gov/index.html). The studies involving human participants were approved by the national cancer institution. Written informed consent for participation was not required for this study in accordance with national legislation and institutional requirements. Enzhao Zhu had full access to all the data in the study and takes responsibility for the integrity of the data and the accuracy of the data analysis.
