# Supplementary material for: Quantified treatment effect at the individual level is more indicative for personalized radical prostatectomy recommendation: implications for prostate cancer treatment using deep learning
Source: J Cancer Res Clin Oncol. 2024 Feb 1;150(2):67. doi: 10.1007/s00432-023-05602-4 (PMC10834597; doi:10.1007/s00432-023-05602-4)
Supplement: Supplementary file 1 — Supplementary file1 (DOCX 16 KB) [file 432_2023_5602_MOESM1_ESM.docx]

Table S1: Impact on integrated Brier score of SNB after exclusion of variables

| Excluded variables | Overall IBS | IBS^a^ | IBS^b^ |
| --- | --- | --- | --- |
| None | 0.05 (0.04–0.05) | 0.10 (0.09–0.12) | 0.04 (0.03–0.04) |
| Grade IV | 0.16 (0.13–0.17) | 0.25 (0.23–0.26) | 0.13 (0.12–0.14) |
| Gleason score 10 | 0.13 (0.12–0.13) | 0.22 (0.22–0.23) | 0.11 (0.10–0.11) |
| Lung metastasis | 0.13 (0.11–0.15) | 0.21 (0.20–0.23) | 0.11 (0.10–0.11) |
| Gleason score 4 | 0.09 (0.08–0.10) | 0.18 (0.17–0.19) | 0.08 (0.08–0.09) |
| Gleason score 5 | 0.10 (0.10–0.11) | 0.18 (0.17–0.18) | 0.08 (0.07–0.10) |
| Liver metastasis | 0.08 (0.07–0.08) | 0.16 (0.15–0.17) | 0.07 (0.07–0.07) |
| Gleason score 2 | 0.06 (0.04–0.07) | 0.14 (0.13–0.15) | 0.05 (0.04–0.06) |
| Tumor size | 0.08 (0.07–0.08) | 0.14 (0.13–0.16) | 0.06 (0.06–0.07) |

Overall IBS, the integrated Brier score of factual survival predication; IBS^a^, integrated Brier score in control group; IBS^b^, integrated Brier score in prostatectomy group.
